# Investigating the effect of independent, blinded digital image assessment on the STOP GAP trial

**DOI:** 10.1186/s13063-017-1779-9

**Published:** 2017-02-02

**Authors:** Emily Patsko, Peter J. Godolphin, Kim S. Thomas, Trish Hepburn, Eleanor J. Mitchell, Fiona E. Craig, Philip M. Bath, Alan A. Montgomery

**Affiliations:** 1 Nottingham Clinical Trials Unit, University of Nottingham, Nottingham, UK; 2 Stroke Trials Unit, Division of Clinical Neuroscience, University of Nottingham, Nottingham, UK; 3 Centre of Evidence Based Dermatology, University of Nottingham, Nottingham, UK; 4 Department of Dermatology, Aberdeen Royal Infirmary, Aberdeen, UK

**Keywords:** Adjudication, Outcome assessment, Blinding, Digital photographs, Digital images, Randomised controlled trial, Clinical trials, Pyoderma gangrenosum

## Abstract

**Background:**

Blinding is the process of keeping treatment assignment hidden and is used to minimise the possibility of bias. Trials at high risk of bias have been shown to report larger treatment effects than low-risk studies. In dermatology, one popular method of blinding is to have independent outcome assessors who are unaware of treatment allocation assessing the endpoint using digital photographs. However, this can be complex, expensive and time-consuming. The objective of this study was to compare the effect of blinded and unblinded outcome assessment on the results of the STOP GAP trial.

**Methods:**

The STOP GAP trial compared prednisolone to ciclosporin in treating pyoderma gangrenosum. Participants’ lesions were measured at baseline and at 6 weeks to calculate the primary outcome, speed of healing. Independent blinded assessors obtained measurements from digital photographs using specialist software. In addition, unblinded treating clinicians estimated lesion area by measuring length and width. The primary outcome was determined using blinded measurements where available, otherwise unblinded measurements were used (method referred to as trial measurements).

In this study, agreement between the trial and unblinded measurements was determined using the intraclass correlation coefficient (ICC). The STOP GAP trial’s primary analysis was repeated using unblinded measurements only. We introduced differential and nondifferential error in unblinded measurements and investigated the effect on the STOP GAP trial’s primary analysis.

**Results:**

Eighty-six (80%) of the 108 patients were assessed using digital images. Agreement between trial and unblinded measurements was excellent (ICC = 0.92 at baseline; 0.83 at 6 weeks). There was no evidence that the results of the trial primary analysis differed according to how the primary outcome was assessed (*p* value for homogeneity = 1.00).

**Conclusions:**

Blinded digital image assessment in the STOP GAP trial did not meaningfully alter trial conclusions compared with unblinded assessment. However, as the process brought added accuracy and credibility to the trial it was considered worthwhile.

These findings question the usefulness of digital image assessment in a trial with an objective outcome and where bias is not expected to be excessive. Further research should investigate if there are alternative, less complex ways of incorporating blinding in clinical trials.

**Trial registration:**

Current Controlled Trials, www.isrctn.com ISRCTN35898459. Registered on 26 May 2009.

**Electronic supplementary material:**

The online version of this article (doi:10.1186/s13063-017-1779-9) contains supplementary material, which is available to authorized users.

## Background

Blinding is the process of keeping treatment assignment hidden after allocation and is used to minimise the possibility of selection, performance and detection biases [1–4]. For this reason, it is considered to be best practice in clinical trial design [5, 6]. A lack of blinding is recognised as a limitation and several issues can arise. If participants are aware of their treatment assignment, their response to subjective outcome measures and cooperation may be influenced, for example when completing a questionnaire [1, 7, 8]. Participants receiving a new intervention may have raised expectations or apprehensions, whilst those receiving standard care may feel relieved or disappointed [1, 8]. An example of performance bias would be unblinded clinicians monitoring participants on a new treatment more closely, or transferring their attitudes about either treatment to the participants. Outcome assessors who are not blinded to treatment allocation may report biased outcomes if they favour a particular intervention, resulting in detection bias. However, this situation is reduced when assessing objective outcomes [1, 7, 8].

Once bias has been introduced in a trial, no analytical techniques can be implemented to reverse its effects. Insufficiently blinded trials have been shown to report larger treatment estimates than blinded studies [9]; a systematic review of 1346 randomised controlled trials (RCTs) found that unblinded trials overestimated the treatment effect by 25% when the outcome was subjective and by 9% when the outcome was objective [10]. A recent Cochrane review by Ndounga Diakou et al. [11] suggested that open-label trials could benefit from blinded outcome assessment to avoid detection bias. In dermatology, the use of digital photographs to assess outcomes is becoming increasingly popular due to the visibility of disease on the skin’s surface and has been used in several RCTs [9, 12, 13]. Digital images can be sent for assessment to external individuals who have no knowledge of treatment allocation, thus introducing a level of blinding and potentially preventing differential outcome assessment. Additionally, the same assessment team can be used throughout, ensuring consistency.

However, digital photography as a means of outcome assessment can add a layer of complexity to a trial and comes with its own limitations. Purchasing the equipment and software required can be expensive, although cost has decreased recently with the increasing availability of technology [1]. The process can also be time-consuming. Digital images are often required to adhere to a specific set of regulations to ensure consistency, and these are then processed or reformatted for use in image analysis software. Furthermore, images of insufficient quality may need to be retaken, and additional resource must be allocated to the training of clinicians to enable them to take and process the photos in a correct and consistent manner [1, 6]. Similarly, independent assessors require training to utilise image-assessment software.

The objective of this study was to investigate what effect independent, blinded digital image assessment had on the primary outcome of the STOP GAP (Study of Treatments fOr Pyoderma GAngrenosum Patients) trial and to establish whether it offered any protection against detection bias.

## Methods

### The STOP GAP trial

STOP GAP was a multicentre, parallel-group RCT which evaluated the safety and efficacy of the two most commonly used systemic treatments, prednisolone and ciclosporin, in treating pyoderma gangrenosum (PG), a painful, ulcerating skin condition. The primary outcome was speed of healing over 6 weeks for a single target lesion and was chosen as previous work in patients with venous leg ulcers suggested this was a good surrogate for subsequent healing [14]; the STOP GAP trial team’s justification for the use of speed of healing as the primary outcome is given here [15]. The target lesion was identified as being ‘the largest lesion that could be photographed on a single plane’ [13]. The protocol and main results for the STOP GAP trial have been described in detail elsewhere [13, 16]. The trial concluded there was no difference in speed of healing between the two treatments, with an adjusted difference in means of 0.003 (*p* = 0.97, 95% CI −0.20 to 0.21).

### Digital images and assessment methods

Participants visited a clinic at baseline, at 2 and 6 weeks’ follow-up, and when the lesion had healed (up to a maximum of 6 months). At each visit a clinician measured the lesion’s maximum perpendicular width and maximum longitudinal length; the lesion area was then estimated using the formula: length (cm) × width (cm) × 0.785, which approximates to the area of an ellipse (method referred to as unblinded measurements).

It was not possible for clinicians or participants to be blinded due to the noticeable differences in appearance, dosing schedule and side-effect profile of the two drugs. For this reason, digital photographs were taken of the lesion at baseline and at 6 weeks and were evaluated by two independent, blinded assessors.

The two assessors were administrators working for the University of Nottingham within the Centre of Evidence Based Dermatology, and their only involvement in the trial was carrying out the digital image assessment. They were trained by a consultant dermatologist (FEC) and had to work through a series of training images and judged to be competent before being able to undertake assessment of images for the trial. The assessors had a set protocol to follow and carried out their assessments separately, without knowledge of treatment allocation.

**Fig. 1:**
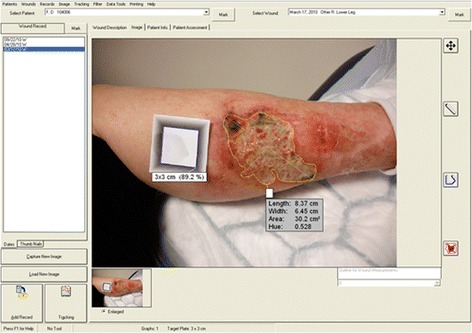
Using specialist software to measure a lesion’s area in the STOP GAP trial

Clinicians were asked to take four photographs of the target lesion at each visit. A target plate was photographed next to the lesion as a point of reference for calibration in the image analysis software (Fig. 1). Photographs were sent to Nottingham Clinical Trials Unit where the best image from each visit was chosen by the trial manager (EJM), who was also blinded to treatment allocation. Sites were contacted to request unsuitable photographs be retaken. Suitable images were resized and the quality adjusted if necessary. These were uploaded to the VERG Videometry VeV (Verge Videometer) MD software (Vista Medical, Winnipeg, MB, Canada) and the target plate was orientated to 3 × 3 cm using its inner border. If orientation failed, these processing steps were repeated with the other photographs to see if an alternative image worked. If all images failed, the site was contacted to request the photographs be retaken. Otherwise the assessors were notified that the images were ready to be measured.

The assessors used the VeV software to trace the circumference of the lesion and obtain a measurement of the lesion area (method referred to as blinded measurements), which is taken to be the ‘gold standard’ (Fig. 1). Blinded measurements may not have been obtained for a participant if no image was available or if the image was of poor quality and further photographs were not available. In addition, two dermatologists independently reviewed the images to ensure that the lesions were consistent with a diagnosis of PG.

**Fig. 2:**
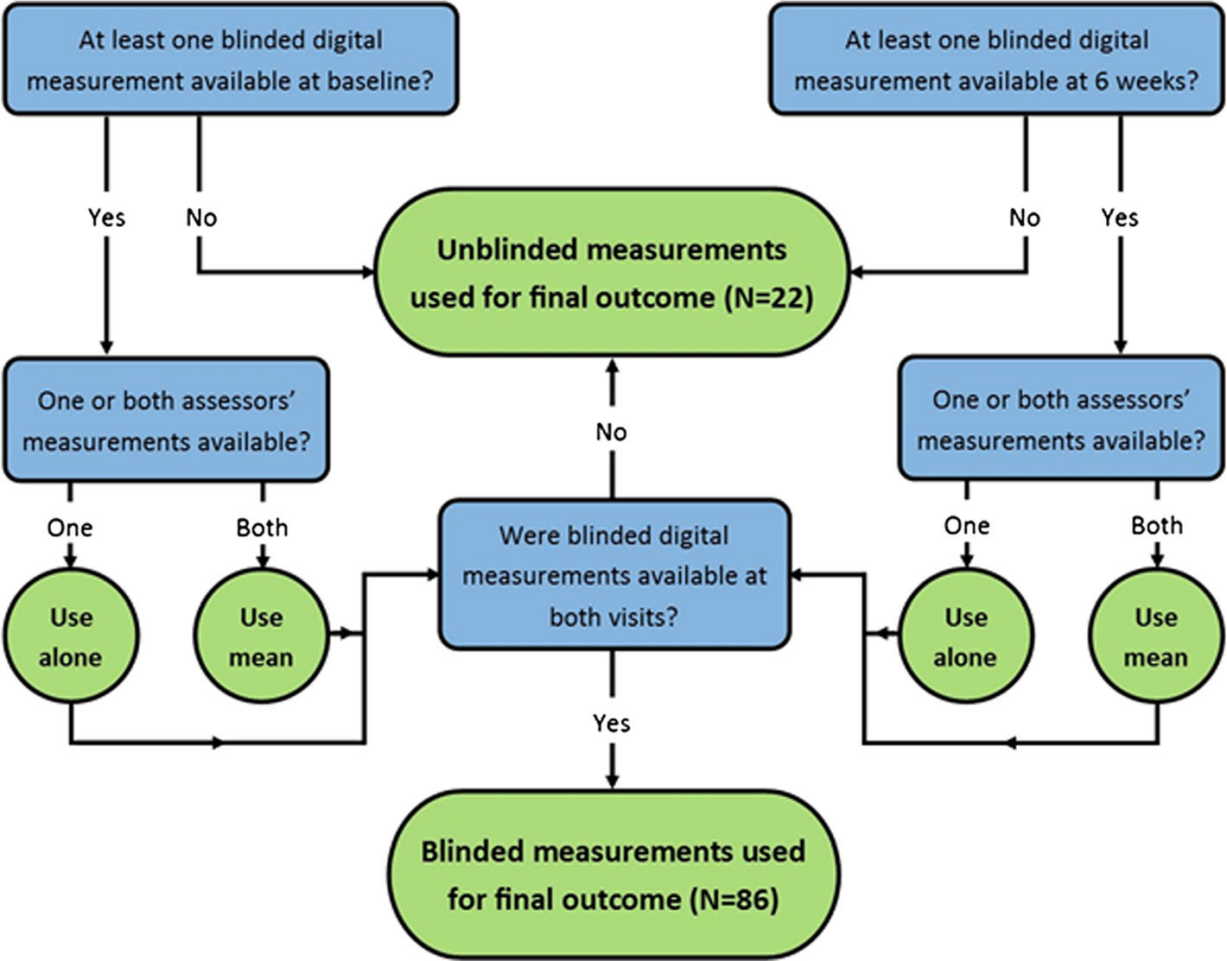
Flow diagram showing criteria for choice of assessment method used in determining speed of healing in the STOP GAP trial analysis

In the STOP GAP trial’s primary analysis, a mixture of the two measurement methods was used (method referred to as trial measurements). For each participant, at least one of the two assessors’ blinded digital measurements had to be available at both baseline and 6 weeks for that method to be used to calculate speed of healing. If both assessors obtained measurements for an image, the mean of the measurements was taken. For participants without blinded measurements at *both* baseline and 6 weeks, their unblinded physical measurements from both visits were used. Figure 2 illustrates this process.

### Statistical analysis

Variables were described as mean (standard deviation) or median (interquartile range) when continuous, and *N* (%) when categorical. Distributions of continuous variables were checked to determine the most appropriate statistic to use for description in each case. Observed agreement between unblinded and trial measurements was assessed using a two-way mixed-effects model intraclass correlation coefficient (ICC), mean difference and mean absolute difference which ignored the direction of difference. Differences were calculated as unblinded measurement minus trial measurement. Agreement on image usability between blinded assessors was evaluated using binary Cohen’s kappa and observed agreement on lesion measurement between blinded assessors was evaluated using two-way mixed-effects model ICC.

To repeat the trial primary analysis speed of healing was calculated as (6-week lesion area minus baseline lesion area in cm^2^)/time in days. The between-arm difference in speed of healing was estimated using a normal linear regression model with baseline lesion size and presence of underlying systemic disease at baseline as covariates. This analysis was first carried out using trial measurements, to replicate the results of the STOP GAP trial analysis [13], and then using unblinded measurements only. The treatment estimates were compared using a test of homogeneity, in which we tested the null hypothesis that the coefficients were equal.

We then increased the observed difference between unblinded and trial measurements (unblinded minus trial) by multiplying it by a scale factor of (a) 2 and (b) 5, in order to investigate whether a large error or bias would alter the findings. We did this in both treatment groups and in the ciclosporin arm only to introduce nondifferential and differential measurement error, respectively. The ciclosporin arm was selected due to STOP GAP specifically investigating whether ciclosporin was superior to prednisolone. It was suspected that any detection bias due to unblinded outcome assessment would appear in the ‘experimental’ arm. Speed of healing was remodelled using these altered measurements and the results compared to the STOP GAP trial analysis using a test of homogeneity for each comparison.

We carried out a sensitivity analysis to investigate the effect of removing outliers. An additional exploratory analysis was undertaken using only participants who were assessed with blinded digital measurements in the STOP GAP trial analysis. The aforementioned primary analysis was repeated on this reduced population.

All analyses were performed in Stata version 14.0.

## Results

Of 112 participants included in the modified intention-to-treat population in STOP GAP, 108 (96%) had measurement data at both baseline and 6 weeks, with 4 lost to follow-up before their 6-week visit. Eighty-six (80%) of the 108 were assessed using digital images. The proportions of patients assessed using blinded and unblinded measurements were similar across the treatment groups (Table 1).

**Table 1:** Characteristics of participants in the STOP GAP trial

	Blinded measurements used^a^		Unblinded measurements used	
Characteristic	Ciclosporin (*n* = 45)	Prednisolone (*n* = 41)	Ciclosporin (*n* = 12)	Prednisolone (*n* = 10)
Age at randomisation (years)				
Mean [SD]	57.4 [16.5]	52.6 [14.6]	55.4 [20.0]	47.7 [16.4]
Gender				
Male	14 (31%)	18 (44%)	3 (25%)	3 (30%)
Female	31 (69%)	23 (56%)	9 (75%)	7 (70%)
Ethnicity				
White	42 (93%)	41 (100%)	11 (92%)	10 (100%)
Non-White	3 (7%)	0 (0%)	1 (8%)	0 (0%)
Type of pyoderma gangrenosum				
Classical	40 (89%)	35 (85%)	8 (67%)	10 (100%)
Cribriform	2 (4%)	2 (5%)	2 (17%)	0 (0%)
Peristomal	2 (4%)	2 (5%)	0 (0%)	0 (0%)
Bullous	0 (0%)	1 (2%)	0 (0%)	0 (0%)
Unsure	1 (2%)	1 (2%)	2 (17%)	0 (0%)
Location of lesion				
Arm	2 (4%)	1 (2%)	0 (0%)	0 (0%)
Leg	31 (69%)	24 (59%)	8 (67%)	8 (80%)
Other	12 (27%)	16 (39%)	4 (33%)	2 (20%)
Blinded measurements available at baseline				
One assessor’s measurement available	20 (44%)	18 (44%)	0 (0%)	2 (20%)
Both assessors’ measurements available	25 (56%)	23 (56%)	1 (8%)	2 (20%)
Neither assessors’ measurements available	–	–	11 (92%)	6 (60%)
Blinded measurements available at 6 weeks				
One assessor’s measurement available	28 (62%)	19 (46%)	2 (17%)	1 (10%)
Both assessors’ measurements available	17 (38%)	22 (54%)	1 (8%)	0 (0%)
Neither assessors’ measurements available	–	–	9 (75%)	9 (90%)
Time from baseline visit to ‘6-week’^b^ follow-up visit (days)				
Median (IQR)	46 (42, 49)	42 (42, 47)	49 (44, 51)	44 (41, 49)
(Min, Max)	(23, 59)	(19, 54)	(42, 80)	(40, 71)

All data are *N* (%) unless otherwise indicated

^a^For each participant, blinded digital measurements had to be available at baseline and at 6 weeks in order for this method to be used for that participant in the STOP GAP trial analysis

^b^Participants whose lesion had healed before the scheduled 6-week visit had their visit brought forward

*IQR* interquartile range, *SD* standard deviation

Agreement between unblinded and trial measurements was excellent at baseline, 6 weeks and when used to calculate speed of healing (Table 2). Table 3 shows agreement between digital image assessors on image usability and digital measurements. There was variable agreement between the assessors; their agreement on image usability was poor to fair, but when they did agree on a given image being usable, their agreement on the actual measurement was excellent.

**Table 2:** Agreement between unblinded measurements and trial measurements

	Ciclosporin (*n* = 57)	Prednisolone (*n* = 51)	Total (*n* = 108)
Lesion size at baseline (cm^2^)			
Mean difference [SD]	4.9 [10.2]	6.7 [22.1]	5.8 [16.8]
Mean absolute difference [SD]	5.5 [9.9]	7.0 [22.0]	6.2 [16.7]
ICC (95% CI)	0.97 (0.94 to 0.99)	0.84 (0.73 to 0.91)	0.92 (0.88 to 0.95)
Lesion size at 6 weeks (cm^2^)			
Mean difference [SD]	4.7 [12.4]	5.2 [25.4]	4.9 [19.5]
Mean absolute difference [SD]	4.8 [12.3]	5.3 [25.3]	5.0 [19.5]
ICC (95% CI)	0.92 (0.85 to 0.95)	0.76 (0.61 to 0.85)	0.83 (0.76 to 0.88)
Speed of healing (cm^2^/day)			
Mean difference [SD]	0.00 [0.24]	−0.04 [0.20]	−0.02 [0.22]
Mean absolute difference [SD]	0.10 [0.21]	0.09 [0.19]	0.10 [0.20]
ICC (95% CI)	0.97 (0.95 to 0.98)	0.89 (0.82 to 0.94)	0.96 (0.94 to 0.97)

The differences between measurement methods were calculated by unblinded physical measurement – trial measurement. *CI* confidence interval, *ICC* intraclass correlation coefficient, *SD* standard deviation

**Table 3:** Agreement between digital image assessors

	Baseline (*n* = 112)	6 weeks (*n* = 108)^a^
Agreement on image usability		
Both assessors agree on image usability	71 (63%)	58 (54%)
Cohen’s Kappa (95% CI)	0.23 (0.07 to 0.40)	0.12 (−0.04 to 0.27)
Agreement on blinded digital measurements		
Both assessors’ measurements available	52 (46%)	40 (37%)
ICC of assessors’ measurements (95% CI)	1.00 (1.00 to 1.00)	1.00 (1.00 to 1.00)

All data are *N* (%) unless otherwise indicated

^a^4 patients were lost to follow-up (did not have a 6-week visit)

*CI* confidence interval, *ICC* intraclass correlation coefficient

Independent, blinded digital image assessment made no material difference to the primary outcome for the trial with the treatment estimates and corresponding confidence intervals for trial and unblinded measurements being near identical (Table 4).

**Table 4:** Speed of healing over 6 weeks by assessment method

Lesion size assessment method	Treatment group	Number in group	Mean (SD) speed of healing (cm^2^/day)	Difference in means (ciclosporin –prednisolone)	Adjusted difference^a^ (95% CI)	*p* value	*p* value^b^
Trial measurements	Ciclosporin	57	−0.21 (1.00)	−0.074	0.003 (−0.20 to 0.21)	0.97	1.00
	Prednisolone	51	−0.14 (0.42)				
Unblinded measurements only	Ciclosporin	57	−0.21 (1.00)	−0.035	0.003 (−0.24 to 0.25)	0.98	
	Prednisolone	51	−0.18 (0.47)				

^a^Adjusted by stratification factors baseline lesion size and presence of underlying systemic disease

^b^ *p* value for test of homogeneity

Increasing the magnitude of the observed difference between unblinded and trial measurements in both treatment arms and in the ciclosporin arm only resulted in treatment estimates further from zero, but these did not materially alter the conclusions from the primary analysis in STOP GAP (Table 5).

**Table 5:** Speed of healing over 6 weeks using unblinded measurements only, with increased difference between measurements

Observed measurement difference^a^ increase	Treatment group	Number in group	Mean (SD) speed of healing (cm^2^/day)	Difference in means (ciclosporin –prednisolone)	Adjusted difference^b^ (95% CI)	*p* value	*p* value^c^
Both treatment groups							
2×	Ciclosporin	57	−0.21 (1.05)	0.004	0.017 (−0.29 to 0.32)	0.91	0.93
	Prednisolone	51	−0.21 (0.59)				
5×	Ciclosporin	57	−-0.20 (1.44)	0.122	0.115 (−0.38 to 0.61)	0.65	0.66
	Prednisolone	51	−0.33 (1.11)				
In ciclosporin group only							
2×	Ciclosporin	57	−0.21 (1.05)	−0.071	0.115 (−0.12 to 0.35)	0.34	0.35
	Prednisolone	51	−0.14 (0.42)				
5×	Ciclosporin	57	−0.20 (1.44)	−0.066	0.212 (−0.17 to 0.59)	0.27	0.28
	Prednisolone	51	−0.14 (0.42)				

^a^The differences between measurement methods were calculated by unblinded physical measurement – trial measurement

^b^Adjusted by stratification factors baseline lesion size and presence of underlying systemic disease

^c^ *p* value for test of homogeneity between the treatment estimate and the treatment estimate from the STOP GAP trial analysis using trial measurements (see Table 4)

**Fig. 3:**
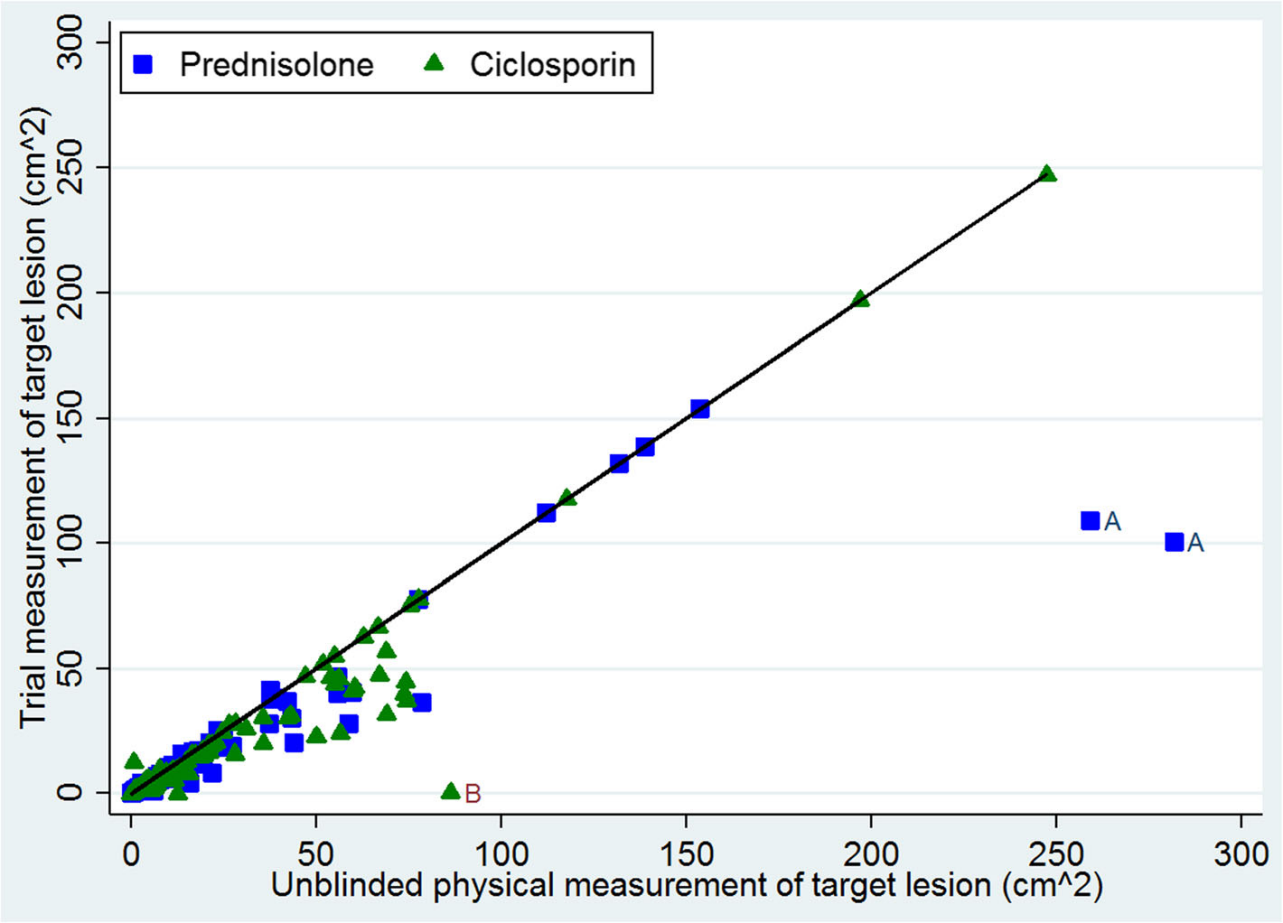
Plot of trial measurements against unblinded measurements at baseline and 6 weeks

There were three unusual observations, as shown in Fig. 3. Participant A in the prednisolone arm had unblinded measurements approximately 150 cm^2^ larger than their blinded measurements at both baseline and 6 weeks. Participant B, allocated to ciclosporin, had a substantial difference between measurement methods at 6 weeks; their blinded measurement was 0.42 cm^2^, whilst their unblinded measurement was 86.35 cm^2^. Removing these two participants improved the agreement between the trial and unblinded measurements (Additional file 1). Repeating the analysis without these participants again did not show a noticeable difference (trial measurements used: adjusted difference = 0.04 (95% CI −0.17 to 0.25, *p* = 0.71), unblinded measurements used: adjusted difference = 0.08 (95% CI −0.14 to 0.29, *p* = 0.47)).

Exploratory analysis was conducted on the 86 participants who were assessed using blinded measurements in STOP GAP. There was a more noticeable difference in the estimated treatment effects than in the primary analysis, but there was insufficient evidence to conclude benefit of either treatment (Additional file 2).

## Discussion

### Summary of main findings

In STOP GAP, unblinded physical measurements consistently overestimated lesion size, but agreement between trial and unblinded measurements was still excellent. This overestimation using unblinded measurements could be explained by the approximation of the lesion area as elliptical in shape. Our study found similar results when using either the blinded or unblinded primary outcome; there was no evidence of detection bias, suggesting that the use of independent, blinded digital image assessment was not necessary in the STOP GAP trial. Increasing the magnitude of the observed difference between unblinded and trial measurements favoured ciclosporin over prednisolone, although precision remained insufficient to rule out benefit of either treatment. These findings question the usefulness of digital image assessment in a trial with an objective outcome and where bias is not expected to be excessive.

### Findings in context of previous research

A recent Cochrane review by Ndounga Diakou et al. [11], which investigated how the blinding status of on-site assessors affected the benefit of independent, blinded outcome assessment, found similar conclusions. The review found that blinded outcome assessment had little to no impact on the treatment-effect estimates. However, as mentioned previously Ndounga Diakou et al. suggest that open-label trials could benefit most from additional blinded assessment. Therefore, it is important to note that as our findings do not appear to agree with this notion, they may not be generalisable. Although, this disagreement may be due to the fact that Ndounga Diakou et al. looked at a range of studies with subjective outcomes, whereas we investigated one trial with an objective primary outcome.

When comparing the use of trial and unblinded measurements when determining the STOP GAP trial’s primary outcome we found the estimated treatment effects to be identical, contrary to other studies [17–19] which have found unblinded trials often overestimate treatment effects. However, this may be partially attributed to the fact that our primary analysis did not directly compare blinded versus unblinded outcome assessment; only 80% of the trial measurements were blinded. The exploratory analysis, using the reduced population of participants assessed using blinded measurements, found the treatment-effect estimate using the unblinded measurements to be at least ten times larger in magnitude than when only the blinded measurements were used. However, this analysis still resulted in the same conclusion as our primary analysis in that digital image assessment did not change the trial findings.

The potential for bias tends to be higher when the primary outcome is a subjective measure, such as quality of life, rather than a clearly defined objective one [1, 5, 20, 21] such as lesion size in STOP GAP. Moreover, the overestimation in the unblinded physical measurements was consistent over both treatment groups rather than biased, and was likely due to their crude nature. Additionally, there is no recommended initial treatment nor were there any preconceived ideas about the superiority of either drug due to PG being very rare. This could explain why we observed no difference between assessment methods, as nondifferential error can be expected, which would dilute the treatment estimates rather than introduce bias [22].

A recent study [1], which reviewed the use of digital photographs for blinded outcome assessment in a clinical trial looking at treatments for verrucae [23], found that blinded digital image assessment did not have an impact on the trial conclusions. Similarly, the conclusions of STOP GAP would not have been altered even if the observed difference between the unblinded and trial measurements was increased differentially in the ciclosporin arm by a factor of 5. However, this may be due to the fact that the speed of healing was already very similar between the two treatment groups in the trial, so it would be hard for detection bias to introduce enough variation to change the result. If the trial initially provided stronger evidence of a treatment effect, then our study might have reached a different conclusion.

It is important to consider the cost of digital image assessment, which we estimated to be £20,000, approximately 2% of the total budget for this trial. This includes the cost of equipment and software, training of both image assessors, travel for the specialist trainers, and payment for all staff involved to carry out the image processing and assessment. Alongside costs, it is also important to recognise the time involved in these processes. For instance, whilst the unblinded assessors found the measuring process relatively straightforward, the blinded image assessors had difficulties initially using the software and found the actual measurement process to be time-consuming.

Additionally, in STOP GAP, there was a difficulty in measuring photographs of lesions that were particularly large or circumferential such as when stretched around the curvature of a limb. This finding agrees with a study [24] that compared wound measurement using two techniques; a manual tracing process and computer software which calculated the measurement after the wound was photographed. The study found that as digital photographs are a 2D image attempting to capture a 3D structure, ‘discrepancy may also occur when tracing circumferential wounds’. It can also be hard to measure digital photographs of lesions when a participant exhibits subtle symptoms, such as redness or swelling, which can affect outcome measures. In fact, the outlying participants referred to earlier as A and B are such cases of this. An inspection of their digital images revealed that participant A had a large circumferential lesion which covered most of their forearm, whilst participant B’s lesion was healing in patches with a large amount of surrounding redness. It is probable that these properties were the cause of the disparity between their blinded and unblinded measurements.

Furthermore, blinded digital measurements were only obtained for 80% of patients; 20% of the sample would have had no primary outcome data if unblinded physical measurements had not also been taken as a back-up and so would have been excluded from the primary analysis. An alternative method of incorporating blinding in STOP GAP would have been to use a ‘double dummy’ design [25], with each participant receiving one placebo and one active treatment. However, whilst participants and assessors may be blinded at baseline, this approach would not mask the difference in side effects that would be evident a short time after receiving either treatment. This would have the potential to lead to unblinded participants which, in turn, could lead to unblinded assessors. Additionally, this design is potentially more expensive to implement than digital image assessment. Another possible approach to facilitate blinding could include the use of an additional dermatologist in participant follow-up visits. This dermatologist would be employed purely to conduct measurements, without any further participant interaction or exposure to participant data. However, whilst this would have avoided the complications of digital photography, this approach may not have been feasible in a trial involving a rare disease such as the STOP GAP trial. Moreover, the use of digital image assessment may have increased the accuracy of the measurements as crude physical measurements have been seen to overestimate wound area by 10% [26]. This is desirable regardless of the fact that the results remained unaffected. It also enabled global assessment of the lesion severity and made it possible for experts to check the diagnosis, which was important for a rare condition that recruiting physicians rarely see.

We found that the agreement on measurements between the two assessors was high at both baseline and 6 weeks. As blinded measurements were obtained using computerised assessment and a clear set of instructions, this was not unexpected. The implication from this finding is that the cost of assessment could have been reduced by having only one assessor. However, we observed that if this was the case and only one assessor had been used, on average 49% of the participants would have been assessed using blinded measurements, rather than the 80% observed in STOP GAP. This is due to the low agreement between assessors on image usability and provides some justification for the use of multiple assessors; if digital image assessment is seen to be an important element of the trial design to facilitate blinding, it is vital to ensure that the majority, if not all, of the outcome data received are of a blinded nature. Additionally, the use of multiple assessors adds to the validity of the measurements; if only one assessor is used and is consistently measuring the images incorrectly, there would be no verification and their measurements could cause misleading results and conclusions to be drawn.

An additional benefit that independent, blinded digital image assessment had on STOP GAP was that it improved the credibility of the trial findings. Furthermore, it ensured that the trial would be scored as being of high quality in any subsequent systematic reviews [27]. Due to blinded outcome assessment being seen as the ‘gold standard’, researchers often strive to ensure that at least the primary outcome is blinded. In fact, a study by Olson et al. [28] has shown that manuscripts which report on trials with some form of blinding are three times more likely to be published than those that could have been blinded but were not. Therefore, whilst we have shown in this single case that blinded outcome assessment did not impact on the trial results, it would be of interest to know whether journal editors or the wider scientific community would have accepted the findings had blinded digital image assessment of the primary outcome not been implemented.

### Strengths and limitations

A limitation of this study was that comparing blinded and unblinded assessment was confounded by the measurement method. In order to make a direct comparison, we would have required blinded assessors in clinics taking physical measurements and/or unblinded assessors calculating lesion area using digital images and the specialist software. Furthermore, we were restricted by the small dataset that we had available. As PG is a very rare disease and our analysis was limited to 108 participants, exploring digital image assessment in this setting should be treated as a hypothesis-generating process rather than a hypothesis-confirming one. Therefore, caution should be taken before our results are generalised to other situations or disease areas. However, we have shown that in some circumstances, blinded outcome assessment may not be a necessity to preserve trial quality. We understand that another constraint of our research is that we have only investigated a single trial. Regardless, our findings can be used together with other studies to add to the pool of current knowledge.

One strength of this study is that our results remained robust to a variety of assumptions. With observed difference between unblinded and trial measurements increased both nondifferentially and differentially by up to five times, our primary analysis still suggested that blinded outcome assessment was not necessary in STOP GAP. In fact, we found that it would require the observed difference to be increased by more than 20 times to meaningfully shift the primary outcome, which we feel is implausible. Furthermore, two sensitivity analyses were performed: excluding extreme observations, and an exploratory analysis. Both analyses concluded that digital image assessment did not have an impact on the primary outcome. This helps to reinforce the robustness of our results.

## Conclusions

This study found that independent, blinded digital image assessment did not meaningfully shift trial conclusions in STOP GAP, even when large differential error was introduced. An estimate suggested that the process of collecting and analysing digital images cost approximately 2% of the total trial budget. Given the added accuracy, confidence and credibility that independent image assessment provided in the STOP GAP trial, we conclude that it was worth the minimal expense. We recommend that digital image assessment may be more useful for easily photographed illnesses, where circumferential lesions are not an issue, for example. We advise that future trials which choose to use digital image assessment ensure they have a back-up assessment method in place for when images fail.

Further research should explore the circumstances where digital image assessment may be of most use and if there are alternative, less complex ways of incorporating blinding in clinical trials.

## Additional files

Additional file 1: Agreement between unblinded measurements and trial measurements with outliers removed. Table showing agreement between unblinded measurements and trial measurements with outliers removed. (DOCX 12 kb)
Additional file 2: Exploratory analysis – speed of healing over 6 weeks by assessment method. Table showing results of exploratory analysis; speed of healing over 6 weeks by assessment method. (DOCX 12 kb)

## Abbreviations

ICC: Intraclass correlation coefficient
PG: Pyoderma gangrenosum
RCT: Randomised controlled trial
STOP GAP: Study of Treatments fOr Pyoderma GAngrenosum Patients
VeV: Verge Videometer
