# Supplementary material for: Investigating the effect of independent, blinded digital image assessment on the STOP GAP trial
Source: Trials. 2017 Feb 2;18:53. doi: 10.1186/s13063-017-1779-9 (PMC5288857; doi:10.1186/s13063-017-1779-9)
Supplement: Additional file 1: — Agreement between unblinded measurements and trial measurements with outliers removed. Table showing agreement between unblinded measurements and trial measurements with outliers removed. (DOCX 12 kb) [file 13063_2017_1779_MOESM1_ESM.docx]

**Additional file 1:**

**Table S1: Agreement between unblinded measurements and trial measurements with outliers removed**

|  | **Ciclosporin**  **(n=56)** | **Prednisolone (n=50)** | **Total**  **(n=106)** |
| --- | --- | --- | --- |
| **Lesion size at baseline (cm^2^)**  Mean difference [sd]  Mean absolute difference [sd]  ICC (95% CI) | 4.3 [9.2]  4.9 [8.9]  0.98 (0.95 to 0.99) | 3.9 [8.4]  4.1 [8.3]  0.96 (0.92 to 0.98) | 4.1 [8.8]  4.6 [8.6]  0.97 (0.95 to 0.98) |
| **Lesion size 6 weeks (cm^2^)**  Mean difference [sd]  Mean absolute difference [sd]  ICC (95% CI) | 3.2 [5.8]  3.3 [5.7]  0.98 (0.95 to 0.99) | 1.6 [3.8]  1.8 [3.7]  0.99 (0.97 to 0.99) | 2.5 [5.0]  2.6 [4.9]  0.98 (0.96 to 0.99) |
| **Speed of healing (cm^2^/day)**  Mean difference [sd]  Mean absolute difference [sd]  ICC (95% CI) | -0.02 [0.19]  0.08 [0.17]  0.98 (0.97 to 0.99) | -0.05 [0.17]  0.08 [0.16]  0.92 (0.86 to 0.95) | -0.03 [0.18]  0.08 [0.16]  0.97 (0.96 to 0.98) |

The differences between measurement methods were calculated by unblinded physical measurement – trial measurement.
