# Supplementary material for: Investigating the effect of independent, blinded digital image assessment on the STOP GAP trial
Source: Trials. 2017 Feb 2;18:53. doi: 10.1186/s13063-017-1779-9 (PMC5288857; doi:10.1186/s13063-017-1779-9)
Supplement: Additional file 2: — Exploratory analysis – speed of healing over 6 weeks by assessment method. Table showing results of exploratory analysis; speed of healing over 6 weeks by assessment method. (DOCX 12 kb) [file 13063_2017_1779_MOESM2_ESM.docx]

**Additional file 2:**

**Table S2: Exploratory analysis - speed of healing over six weeks by assessment method**

| **Lesion size assessment method** | **Treatment group** | **No. in group** | **Mean (SD) speed of healing (cm^2^/day)** | **Difference in means**  **(ciclosporin –prednisolone)** | **Adjusted difference^1^**  **(95% C.I.)** | **p-value** | **p-value^2^** |
| --- | --- | --- | --- | --- | --- | --- | --- |
| Blinded digital measurements only | Ciclosporin  Prednisolone | 45  41 | -0.07 (0.26)  -0.06 (0.17) | -0.006 | -0.003 (-0.10 to 0.09) | 0.95 | 0.45 |
| Unblinded physical measurements only | Ciclosporin  Prednisolone | 45  41 | -0.06 (0.23)  -0.11 (0.31) | 0.043 | 0.042 (-0.08 to 0.16) | 0.48 |  |

^1^ Adjusted by stratification factors baseline lesion size and presence of underlying systemic disease.

^2^ p-value for test of homogeneity.
